# Functional outcomes of glansectomy to treat localised penile cancer: a systematic review

**DOI:** 10.1038/s41443-025-01062-1

**Published:** 2025-04-15

**Authors:** Karl H. Pang, Hussain M. Alnajjar, Asif Muneer

**Affiliations:** 1 Male Genital Cancer Centre, Department of Andrology, University College London Hospitals (UCLH) NHS Foundation Trust, London, UK; 2 Division of Surgery and Interventional Science, University College London, London, UK; 3 Department of Urology, Chelsea and Westminster Hospital NHS Foundation Trust, London, UK; 4 National Institute for Health and Care Research (NIHR) Biomedical Research Centre, UCLH NHS Foundation Trust, London, UK

**Keywords:** Surgery, Urogenital diseases

## Abstract

Glansectomy with or without a neoglans reconstruction is commonly performed for invasive penile cancer confined to the glans penis. The aim of penile-preserving procedures is to maintain sexual and urinary function without compromising oncological outcomes. A systematic review was performed to evaluate the functional outcomes following glansectomy. Overall, 14 studies which included 327 glansectomy procedures were analysed. At a mean follow-up of 40.7 (range, 13–52) months, the recurrence rate was 9.1% (0–25%) and the disease-specific survival rate was 87.5–100%. Partial graft loss and meatal stenosis occurred in 6.1% (0–17.6%) and 8.1% (0–14.3%) respectively. 91.1% (50–100%) had preserved erectile function and 62.5% (33.3–100%) were sexually active. 75.6% (66.7–100%) of patients were voiding whilst standing up and 83.7% (63.6–91.2%) had maintained glans sensation. Satisfaction with the overall appearance was achieved in 86.3% (68.2–100%). The reporting of functional outcomes was heterogenous with a limitation that there are no standardised guidelines on the assessment of functional outcomes following glansectomy. Further research should focus on identifying appropriate tools for reporting functional outcomes following glansectomy and standardising reporting.

## Introduction

Penile cancer (PeCa) is a rare male genital cancer with an incidence of approximately 37,699 cases in 2022, representing 0.2% of all cancer sites according to the 2022 GLOBOCAN data [1]. In localised PeCa confined to the glans, penile-preserving treatment such as laser, glans resurfacing or glansectomy are available. The treatment depends on the stage of the disease [2, 3]. In clinical tumour stage 2 (cT2) PeCa, and where in doubt, an MRI excludes the involvement of the distal corpus cavernosa (cT3), a glansectomy is an option [2].

The aim of penile-preserving surgery is to maintain penile length for sexual intercourse and to enable voiding whilst standing up without compromising oncological outcomes. In addition, reconstruction with a split thickness skin graft (SSG) to form a neoglans improves the aesthetic outcomes [4].

The data show that salvage surgery for positive surgical margins (PSM) or local recurrence (LR) is up to 8.3% [5]. In addition, following glansectomy, 95–100% of patients reported good cosmetic outcomes and 50–100% reported normal erections [5]. There is a lack of data focusing solely on functional outcomes including sexual and urinary outcomes and quality of life (QoL). Therefore, the aim of this systematic review was to provide a contemporary update on sexual and urinary outcomes, as well as QoL following glansectomy for PeCa.

## Methods

The systematic review was registered with PROSPERO (CRD42025632864) and was conducted with reference to the 2020 PRISMA statement (Supplementary Table 1) [6]. The search of MEDLINE/PubMed and EMBASE (using OVID) was performed on 29/09/2024. All articles published up until 29/09/2024 were included for screening. The Cochrane library was also searched for any registered trials. The search terms used included (glansectomy OR penile-preserving surgery OR organ-sparing surgery) AND (peni* OR glans) AND (cancer OR malignan* OR tumour OR neoplas*). The population (P), intervention (I), comparison (c), outcome (o), study (s) framework was used to determine the inclusion criteria:

P: adult men with PeCa

I: glansectomy with or without neoglans reconstruction with SSG

C: any forms of treatment for PeCa confined to the glans

O: functional (primary), oncological and quality of life (secondary)

S: any study design including prospective or retrospective observational studies, randomised and non-randomised studies.

Studies with no report on functional outcomes following glansectomy were excluded, as well as non-English and review articles. Case reports, conference abstracts, editorials, letters and commentaries were also excluded. Abstracts, full-text articles, and the reference lists of included articles were screened for eligibility.

### Data extraction and analysis

Data extracted included the study design, number of patients, histopathological data, complications, recurrence and survival rates, sexual and urinary function, and QoL. The risk of bias (Rob) assessment of the included studies was assessed using the JBI checklist [7] (Supplementary Table 2). The screening, data extraction and RoB assessment were performed by 2 authors independently (KHP, HMA) and any disagreements were solved amongst the 2 authors or involvement of the senior author (AM). Where feasible, a pooled analysis was performed calculating the percentage of an event occurring post-surgery such as local recurrence or meatal stenosis. The percentage range was also reported. Where only the median and interquartile ranges were reported, an estimation of the mean was performed using Abbas et al’s online convertor [8]. The mean age and follow-up were calculated by summing all the scores and then dividing by the total number of scores. For the percentage of an event (i.e., local recurrence or meatal stenosis), the number of patients who experienced an event across all the studies was summed and divided by the total number of patients who underwent surgery in all the studies.

## Results

**Fig. 1:**
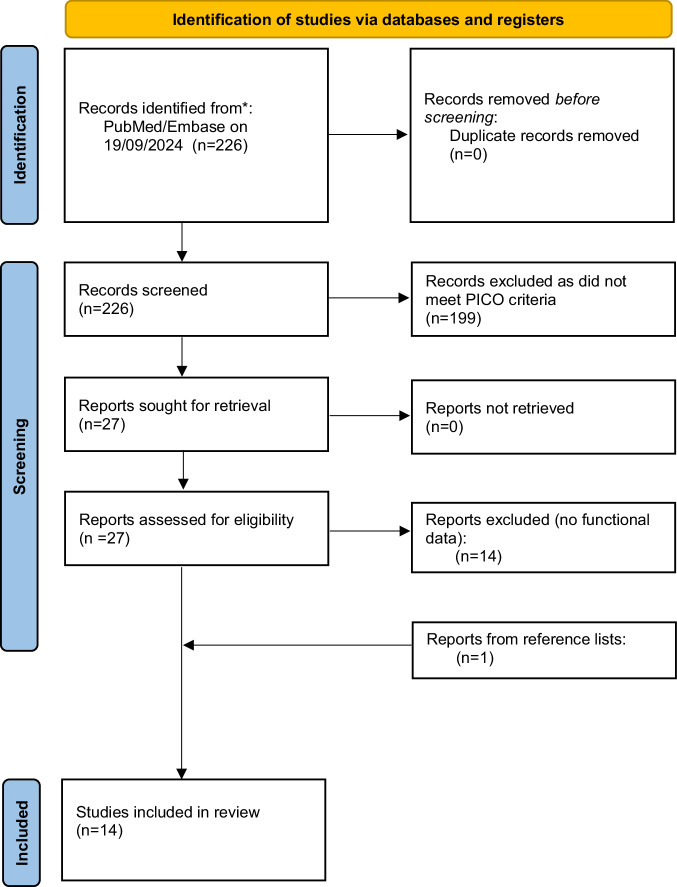
PRISMA 2020 flow chart for the current systematic review.

Overall, 240 articles were identified from the search. Following abstract and full-text article screening as well as reviewing the reference lists of the included articles, a total of 14 studies [9–22], which included 327 glansectomies were analysed (Fig. 1). The RoB assessment of each cohort study demonstrated relatively low risk of bias across most domains, except for the “confounding factors” domain (Supplementary Table 2).

The mean age was 62.7 (51–67) years, and the mean follow-up was 40.7 (13–52) months (Table 1). Procedures performed included partial glansectomy or total glansectomy with or without distal corporectomy or neoglans reconstruction with SSG.

**Table 1:** Baseline characteristics and oncological outcomes of glansectomy for penile cancer.

Author	Country	Design	No. patients	Follow-up (m), Mean (SD/range)	Age (y), Mean (SD/range)	Histology, n (%)	Recurrence (m), n (%)	Survival, %	Complications, n (%)	Salvage amputation, n (%)
^a^Falcone [9]	Italy	Retro	PPS: 99 Glansectomy: 48	24.1 (25.3)	66.5 (13.6)	pTis: 2 (6.3) pTa: 6 (12.5) pT1a: 11 (22.9) pT1b: 5 (10.4) pT2: 21 (243.8) PSM: 0 (0)	12 (25) TTR mean (SD): 21.4 (23.4)	DSS: 87.5%	Wound infection/breakdown: 4 (8.3) Partial graft loss: 1 (2.1) Complete graft loss: 1 (2.1)	NR
^a^Cilio [10]	Italy	Retro	PPS: 34 Glansectomy: 22	Median: 32.5	66.4 (8.7)	pT1-2: 22 (100)	LR: 2 (9.1) TTR: median, 18.5	NR	NR	2 (9.1)
Falcone [11]	Italy	Retro	34	Median (IQR): 12 (12–41)	Median (IQR): 69 (64–77)	pTis: 1 (2.9) pT1: 17 (50) pT2: 16 (47.1)	LR: 6 (17.6) TTR: median (IQR), 16 (12–41)	1y: OS: 91.2% 1y DSS: 91.2%	Meatal stenosis: 2 (5.8) Wound infection: 2 (5.8) Partial graft loss: 6 (17.6) Graft contracture: 1 (2.9)	7 (20.6)
^a^Croghan [12]	Ireland	Retro	33 Partial: 15 Radical: 18	NR	61 (31–79)	pTis: 3 (8.6) pT1: 13 (37.1) pT2: 15 (42.8) pT3: 4 (11.4)	LR: 1 (3.0)	DSS: 100%	NR	NR
Beech [13]	Canada	Retro	12	Median (range): 14 (1–59)	Median (range): 62 (32–85)	pT0: 2 (16.7) pTis: 2 (16.7) pT1a: 5 (41.7) pT1b: 1 (8.3) pT2: 2 (16.7) PSM: 2 (16.7)	LR: 2 (16.7) TTR: NR	OS: NR DSS: NR DFS: 91.7%	90-day Grade >2: 0 (0) Graft loss: 0 (0) Meatal stenosis: 1 (8.3)	1 (8.3)
^a^Perez [14]	Multi-centre	Retro	PPS: 57 Glansectomy: 14	Median (IQR): 48.2 (3–143)	Median (IQR): 58.6 (36–90)	pTis: 2 (14.3) pT1: 6 (42.9) pT2: 6 (42.9) PSM: 3 (21.4)	NR	NR	Graft loss: 0 (0) Meatal stenosis: 2 (14.3)	NR
^a^Håkansson [15]	Sweden	Retro	PPS: 27 Glansectomy: 15	Median (range): 10 (1–25)	63.8 (37–78)	pT0: 1 (6.7) pT1: 8 (53.3) pT2: 5 (33.3) pT3: 1 (6.7) PSM: 1 (6.7)	LR: 0 (0) TTR: NR	NR	Graft infection: 2 (13.3)	
Scarberry [16]	USA	Retro	Total: 6 Glansectomy: 2 Glansectomy + distal corporectomy: 4	52 (25–84)	67 (56–87)	NR	NR	NR	NR	NR
^a^Sedigh [17]	Italy	Retro	PPS: 41 Glansectomy: 23	36 (20–50)	60 (45–68)	pT1: 10 (43.5) pT2: 13 (56.5) PSM: 0 (0)	LR: 0 (0) TTR: n/a	OS: NR DSS: NR DFS: 100%	Meatal stenosis: 3 (13.0)	NR
^a^Veeratterapillay [18]	UK	Retro	Total: 62 Glansectomy: 46 Partial glansectomy + STSG: 1 Glansectomy + distal corporectomy: 15	40 (12–72)	62 (32–89)	pTis: 10 (16.7) pT1: 31 (51.7) pT2: 19 (31.7) PSM: 2 (3.2)	LR: 4 (6.5) TTR: 15m	OS: NR DSS: NR	NR specifically for glansectomy	1 (1.6)
O'Kane [19]	UK	Retro	25	28 (6–66)	60 (39–83)	pTis: 6 (24) pT1: 15 (60) pT2: 3 (12) pT3: 1 (4) PSM: NR	LR: 1 (4) TTR: NR	OS: NR DSS: 92%	Graft loss: 0 (0) Meatal stenosis: 2 (8)	1 (4)
^a^Palminteri [20]	Italy	Retro	PPS: 21 Glansectomy: 10	45	NR	pT1: 8 (80) PSM: NR	LR: 0 (0) TTR: n/a	OS: 100% DSS: 100%	Meatal stricture: 1 (10)	NR
Morelli [21]	Italy	Pro	Total: 15 Glansectomy + STSG: 14 Glansectomy + distal corporectomy: 1	36 (10–67)	51 (42–59)	pTa: 2 (13.3) pT1: 7 (46.7) pT2: 4 (26.7) pT3: 2 (13.3) PSM: 0 (0)	LR: 0 (0) TTR: n/a	OS: NR DSS: 93.3%	Partial graft loss: 2 (13.3) Meatal stenosis: 1 (6.7)	N/A
^a^Gulino [22]	Italy	Retro	PPS: 14 Glansectomy: 8	13 (9–14) for all patients	54 (all patients)	pT1: 8 (100) PSM: 0 (0)	LR: 0 (0) TTR: n/a	OS: NR DSS: NR	No postoperative complications	NR

*CIS* carcinoma in situ, *DFS* disease-free survival, *DSS* disease specific survival, *FU* follow up, *LR* local recurrence, *OS* overall survival, *PeIN* penile intraepithelial neoplasia, *Pros* prospective, *Retro* retrospective, STSG split thickness skin graft, *TTR* time to recurrence.

^a^Reported within a penile-preserving surgery (PPS) series which included circumcision, wide local excision, laser or glans resurfacing.

### Functional outcomes

The assessment of functional outcomes was heterogenous with studies using both validated and locally designed non-validated questionnaires (Table 2). Validated questionnaires used included: International Index for Erectile Function (IIEF) [23], Changes in Sexual Function Questionnaire (CSFQ) [24], European Organization for Research and Treatment of Cancer core quality of life questionnaire (EORTC QLQ-C30) [25], EuroQoL questionnaire visual analogue scale (EQ-5D-3L VAS) [26], International Consultation on Incontinence Questionnaire Urinary Incontinence Short Form (ICIQ-UI SF) [27], International Consultation on Incontinence Questionnaire Male Lower Tract Symptoms (ICIQ-MLUTS) [28], Sex Encounter Profile (SEP) [29] and the International Prostate Symptom Score (IPSS) [30].

**Table 2:** Functional outcomes of glansectomy for penile cancer.

Author	Validated questionnaires, n (%)	Non-validated questionnaires, n (%)
^a^Falcone [9]	Change in IIEF-15 at 6–12m mean (SD): +2.8 (7.4) points Change in IIEF-EF at 6–12m mean (SD): +1.3 (8.3) points Change in IPSS at 6–12m mean (SD): −0.3 (−4.1) points	Maintained glans sensation: 14/22 (63.6) Satisfied with appearance: 15/22 (68.2) Negative impact on QoL: 6/22 (27.3) Negative impact on sexual life: 6/22 (27.3) Negative impact on urinary function: 6/22 (27.3)
^a^Cilio [10]	Postop 1y IIEF-5: mean (SD) 17 (3.6) CSFQ mean (SD): Sexual desire/frequency, 7.9 (1.3); Sexual desire/interest, 11.3 (2.4); Arousal/excitement, 9 (2.1); Orgasm/completion, 8 (3.1); Total, 40.3 (2.5)	NR
Falcone [11]	Postop 1y IIEF: −3 points Postop SEP-2 and SEP-3 same as preop IPSS: +4 points ICIQ-UI SF same as preop	Maintained glans sensation: 31/34 (91.2) Satisfied with appearance: 30/34 (88.2) Negative impact on QoL: 4/34 (11.8) Negative impact on sexual life: 3/34 (8.8)
^a^Croghan [12]	EORTC QLQ-C30: *Overall health* Partial: mean 6 Radical: mean 5.7 *Overall quality of life* Partial: mean 5.7 Radical: mean 6 IIEF-5: mean 14.9 (Partial); 15.8 (Radical)	Sexually active: 18/33 (54.6) (Partial, 9/15 (60); Radical, 9/18 (50)) Standing voiding (often/always): 22/33 (66.7) (Partial, 9/15 (60), Radical, 13/18 (72.2) Very happy/happy with voiding: 27/33 (81.8) (Partial, 11/15 (73.3), Radical, 16/18 (88.9%) Satisfied with appearance: 25/32 (78.1) (Partial, 12/15 (80), Radical, 13/17 (76.5) Satisfied with penile length: 17/32 (53.1) (Partial, 6/15 (40), Radical, 11/17 (64.7) Satisfied with shape of glans: 23/32 (71.9) (Partial, 12/15 (80), Radical, 11/17 (64.7) Maintained glans sensation: 27/30 (90) (Partial, 14/14 (100), Radical, 13/16 (81.2)
Beech [13]	NR	Preserved EF: 12/12 (100) Standing voiding: 12/12 (100) Satisfied cosmesis: 12/12 (100)
^a^Perez [14]	EQ-5D-3L VAS: median (IQR) 85% (70-90) IIEF-5: 8 (11–23) ICIQ-MLUTS-voiding: 4 (2–6) ICIQ-MLUTS-QoL: 2 (0–9)	Sexually active: 14/14 (100)
^a^Håkansson [15]	NR	Good/excellent cosmetic and functional results: 15/15 (100)
Scarberry [16]	IIEF-15 mean 3.7 IIEF-EF mean 7.3	Erection with normal rigidity: 3/6 (50) Sexually active: 2/6 (33.3) Sexual symptoms do not at all interfere with daily lives: 5/6 (83.3) Urinary symptoms do not at all interfere with daily lives: 5/6 (83.3) Satisfied with the outcome: 6/6 (100)
^a^Sedigh [17]	Postop IIEF-15: −11.4 points Postop IIEF-EF: −3.3 points Postop SEP-2: −27.3%; SEP-3: −39.2%	Genital sensitivity: reduced in 59.1% patients
^a^Veeratterapillay [18]	NR	Good erections at 1y: 85% Good/excellent cosmetic outcomes: 95%
O'Kane [19]	NR	Achieve erections: 9/11 (81.8) Sexually active: 6/11 (54.6)
^a^Palminteri [20]	NR	Satisfied with aesthetic results: 10/10 (100) Recovered sexual function: 10/10 (100)
Morelli [21]	NR	Preserved EF: 17/17 (100) Reduced glans sensitivity: 15/15 (100)
^a^Gulino [22]	Postop IIEF-EF at 12m: +1 point	Urethral neoglans showed improved thermal, touch and static sensitivity. ^b^Improvements in QoL regarding patient subjective feelings, and relationships with family and partner based on analogue scale.

*CSFQ* changes in sexual function questionnaire, *EF* erectile function, *EORTC QLQ-C30* European organization for research and treatment of cancer core quality of life questionnaire, *EQ-5D-3L VAS* euroQoL questionnaire visual analogue scale, *ICIQ-UI SF* international consultation on incontinence questionnaire urinary incontinence Short Form, *ICIQ-MLUTS* international consultation on incontinence questionnaire male lower tract symptoms, Short Form *IIEF* international index for erectile dysfunction, *PROM* patient-reported outcome measure, *QoL* quality of life, *SEP* sex encounter profile, *IPSS* international prostate symptom score.

^a^Reported within a penile-preserving surgery (PPS) series which included circumcision, wide local excision, laser or glans resurfacing.

^b^Authors used the Bigelow and Young Score.

Overall, 62.5% (33.3–100%) of patients were sexually active at follow-up, 91.1% (50–100%) had preserved erectile function. The change in IIEF-EF score ranged between −3.3 and 1.3 points. Glans sensation was maintained in 83.7% (63.6–91.2%) of patients, and 75.6% (66.7–100%) of patients were able to stand up and void. Satisfaction with the overall appearance or outcomes was achieved in 86.3% (68.2–100%) of patients. Negative impact on QoL and sexual life was reported in 27.3% respectively [9].

### Oncological outcomes and complications

Oncological outcomes and complications are summarised in Table 1. In all, 3.2% (0–21.4%) of patients had a PSM and 9.1% (0–25%) had a recurrence. The disease-specific survival (DSS) was 87.5–100%. Partial graft loss occurred in 6.1% (0–17.6%) of patients [11], and meatal stenosis occurred in 8.1% (0–14.3%) of patients [14]. Salvage surgery for PSM and/or recurrence was required in 7.7% (0–20.6%) of patients [11].

## Discussion

In this systematic review, we focused solely on functional outcomes and QoL following glansectomy for invasive PeCa. From the 14 studies which met the PICOS inclusion criteria, only 5 studies reported on glansectomy per se. The remaining studies reported on a combined series which included other penile-preserving procedures as well as penectomy. With the heterogeneity of the patient cohorts in the studies analysed, data extraction to identify the patients undergoing glansectomy becomes difficult. In addition, most studies primarily focused on oncological outcomes as opposed to functional outcomes. Again this posed difficulties in extracting the relevant functional data. Nevertheless, in this review the reporting and results of functional outcomes were heterogenous. This was partly related to the wide range in the number of patients included in the studies analysed, with the smallest series including 6 patients, and the largest including 62. The assessment of functional outcomes was not standardised and currently there are no standardised reporting system based on the currently available PeCa guidelines [2, 3]. Some studies have used validated questionnaires such as IIEF, IPSS and ICIQ, whilst other studies have used locally-designed questionnaires. It is worth noting, the common validated questionnaires used in the included studies are not specific to post-PeCa surgery, but generic tools to assess outcome measures from urological procedures. Moreover, the IPSS is used primarily to evaluate prostatic symptoms and not for urinary symptoms associated with a neoglans or meatal reconstruction following glansectomy. In studies that utilised the pre- and postoperative IIEF-EF score, 2 studies [9, 22] reported an increase in the score and 1 study [17] reported a decrease in the score, reflecting contradictory data. Reporting a change in scores is a more reliable method in assessing erectile function, as studies just reporting postoperative IIEF or functional outcomes scores without taking into consideration of the preoperative status are not useful. Therefore, future studies should ideally report pre- and postoperative functional parameters with changes in questionnaire scores where appropriate. What is important following penile-preserving surgery, is not the erectile function as such, but whether the residual penile length is sufficient for penetration and whether the patient can void whilst standing up. Therefore, a more reliable outcome measure is to ask whether patients can still penetrate or void whilst standing up following glansectomy. In this review, 75.6% (66.7–100%) of patients were able to void whilst standing up [12, 13]. The proportion of patients who were sexually active varied between 33–100% [14, 16]. Again, this may be associated with the difference in the number of patients and follow-up period in the included studies, as well as individual centre’s expertise.

With regards to postoperative meatal function, as mentioned above, IPSS may not be the best tool to use. Specific assessment should focus on examining the meatus to see if there is any stenosis and asking whether the stream is narrow or if there is any spraying. A uroflowmetry and urethroscopy may be required for a more objective assessment.

In general, good aesthetic outcomes or satisfaction with the cosmetic outcome was achieved in majority of patients. Glansectomy did not affect QoL in most patients, as up to 27.3% of patients reported a negative impact on QoL and sexual life [9, 11]. In addition, Gulino et al. [22] reported improvements in QoL regarding patient subjective feelings, and relationships with family and partner based on Bigelow and Young scores [31].

The individual centre’s surgical experience and slight variation in glans reconstruction may result in the variable graft take rates, meatal complications, cosmetic and functional outcomes observed in this study. High-volume centres where PeCa management is centralized would be expected to have better surgical outcomes [32]. Moreover, the varied tumour stage within the included studies and lengths of follow-up may induce heterogenicity in results.

### Authors’ experience

Glansectomy is now widely accepted as the procedure of choice for invasive cT2 PeCa. We previously reported the outcomes of 172 glansectomies performed in a single centre, and the LR rate was 9.3%, complete or near-complete graft loss rate was 3.4% and meatal stenosis rate was 2.8% [4]. These outcomes are in the lower range compared to data reported in the literature. When performed by experienced surgeons and in high-volume centres, the oncological outcomes are good, complication rates are low and cosmetic outcomes are excellent.

**Fig. 2:**
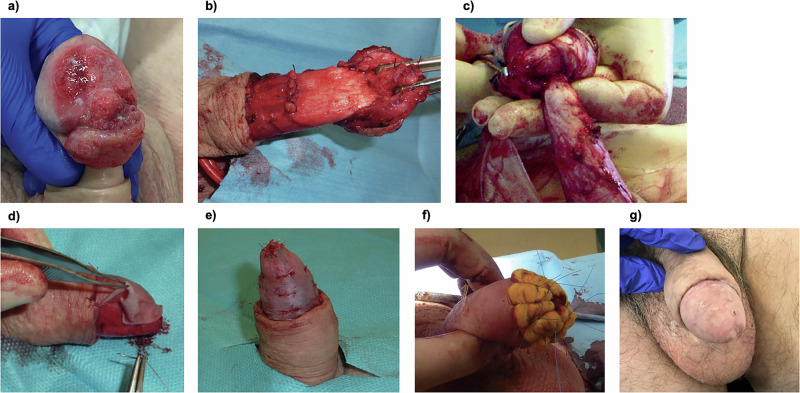
Intraoperative and postoperative images of glansectomy and neoglans reconstruction. **a** tumour localised to the glans penis; **(b**) dissection above the Buck’s fascia; (**c**) transection of the urethra leaving around 1 cm length beyond the corporal tips; (**d**, **e**) a split-thickness skin graft is sutured to the denuded glans penis; (**f**) a tied-over dressing for graft application (TODGA) is applied; (**g**) postoperative appearance.

Our approach to the procedure has been described previously [4] and is summarised below (Fig. 2).

An incision is made on the outer prepuce. If the patient had been circumcised, an incision is made along the circumcision scar. The incision is deepened circumferentially, and a plane is created between above Buck’s fascia and the glans in order to preserve the vascular supply if a graft is planned (Fig. 2b). If a SSG is not planned, especially if the tumour is adherent to Buck's fascia, dissection is performed under the fascia. The glans is dissected off the corporal bodies until the urethra is the only remaining attachment, maintaining ~1 cm length beyond the corporal tips if possible (Fig. 2c). The urethra is transected, spatulated ventrally and is splayed over the corpora cavernosa heads at 2–4 points with 4–0 absorbable suture. Biopsies of the urethral margin and distal corporal bodies are taken. If the distal corporal bodies are involved macroscopically, distal corporectomies are performed. The skin is brought to the urethra with 4–0 absorbable suture. A 14–16Fr 2-way indwelling catheter is left in place for around 5 days [4].

In patients who desire to have a neoglans, a SSG can be used for reconstruction. Commonly, the inner thigh is selected for SSG harvesting using an air-powered dermatome. Graft thickness ranges from 0.008 to 0.018 inches. The donor site is infiltrated with 1% lidocaine and 1:200,000 adrenaline and covered with a calcium alginate dressing. The distal part of the penile shaft skin is sutured to the corporal bodies to leave around 2 cm of the distal corporal bodies exposed as a graft bed for the reconstruction of a neoglans. The SSG is laid onto the distal corporal bodies and is sutured to the edge of the penile shaft skin proximally and to the urethral edge distally with 4–0 and 5–0 absorbable sutures (Fig. 2d, e). Absorbable quilting sutures with monofilament poliglecaprone 5.0 are used. A 14–16Fr 2-way urethral catheter is left insitu for around 7–10 days and a paraffin soaked tie-over dressing for graft application (TODGA) is applied and sutured in place (Fig. 2f) [4, 33].

### Future directions

It is important to establish a standardised system for reporting functional outcomes following PeCa surgery. This would allow a more meaningful statistical pooled analysis to be performed. Standardised reporting may require developing consensus recommendations from international experts or guidelines panels, which may require the development of PeCa specific assessment tools focusing on comprehensive functional metrics.

### Limitations

In an attempt to select high-quality studies, letters, editorials and commentaries were excluded as well as single case reports. This restriction may have omitted important reports on functional outcomes following glansectomy. The assessment tools used were heterogenous amongst the included studies. Moreover, within the IIEF questionnaire, there were 3 different types used which included IIEF-5, IIEF-15 or IIEF-EF. In addition, the reporting was not consistent, for example, some studies reported postoperative IIEF scores, and some reported changes in IIEF score, which made performing a meta-analysis not possible.

## Conclusion

Penile-preserving surgery should be considered in all patients with localised PeCa. Results demonstrated that the functional and cosmetic outcomes following glansectomy is acceptable without compromising oncological outcomes. Further research is required to enable recommendations to be developed on the standard reporting of functional outcomes following glansectomy for PeCa.

## Supplementary information

1 and 2

## Data Availability

All data generated or analysed during this study are included in this published article and supplementary files.
